# *Chlamydia pecorum* prevalence in South Australian koala (*Phascolarctos cinereus*) populations: Identification and modelling of a population free from infection

**DOI:** 10.1038/s41598-019-42702-z

**Published:** 2019-04-18

**Authors:** Jessica Fabijan, Charles Caraguel, Martina Jelocnik, Adam Polkinghorne, Wayne S. J. Boardman, Elisa Nishimoto, Greg Johnsson, Robyn Molsher, Lucy Woolford, Peter Timms, Greg Simmons, Farhid Hemmatzadeh, Darren J. Trott, Natasha Speight

**Affiliations:** 1 School of Animal and Veterinary Sciences, The University of Adelaide, Roseworthy, 5371 South Australia Australia; 2 Faculty of Science, Health, Education and Engineering, University of the Sunshine Coast, Sippy Downs, 4558 Queensland Australia; 3 Kangaroo Island Veterinary Clinic, Kingscote, 5223 South Australia Australia; 4 Department for Environment and Water, Adelaide, 5000 South Australia Australia; 5 School of Veterinary Sciences, The University of Queensland, Gatton, 4343 Queensland Australia

**Keywords:** Bacteriology, Infectious-disease diagnostics

## Abstract

*Chlamydia pecorum* is an established and prevalent infection that produces severe clinical disease in many koala populations, contributing to dramatic population declines. In wild South Australian koala populations, *C*. *pecorum* occurrence and distribution is unknown. Here, *C*. *pecorum-*specific real-time quantitative PCR (qPCR) was applied to ocular and urogenital swabs from targeted surveys of wild koalas from the mainland Mount Lofty Ranges (MLR) (n = 75) and Kangaroo Island (KI) (n = 170) populations. Historical data from 13,081 KI koalas (1997–2018) provided additional evidence for assessing the absence of *C*. *pecorum* infection. In the MLR population, 46.7% (CI: 35.1–58.6%) of koalas were *C*. *pecorum* positive by qPCR but only 4% had grade 3 clinical disease. MLR koala fertility was significantly reduced by *C*. *pecorum* infection; all reproductively active females (n = 16) were *C*. *pecorum* negative, whereas 85.2% of inactive females (n = 23) were positive (P < 0.001). KI koalas were *C*. *pecorum* negative and the population was demonstrated to be free of *C*. *pecorum* infection with 95% confidence. *C*. *pecorum* is a real threat for the sustainability of the koala and KI is possibly the last isolated, large *C*. *pecorum*-free population remaining in Australia. These koalas could provide a safeguard against this serious disease threat to an iconic Australian species.

## Introduction

*Chlamydia pecorum* is recognised as the most significant pathogen causing mortality in koalas and a key contributor to the dramatic population declines in northern Australia (Queensland and New South Wales)^1,2^. *Chlamydia* are obligate intracellular bacteria, of which two species, *C*. *pecorum* and *C*. *pneumoniae*, can infect and cause disease in wild and captive koalas^3^. *C*. *pecorum* is the most pathogenic species, causing conjunctivitis and blindness^4,5^, pneumonia^6^, urinary tract infections (cystitis and nephritis) associated with urinary incontinence^7,8^; and reproductive tract infections resulting in infertility in both females and males due to, paraovarian cysts, endometritis and vaginitis^9^, and orchitis, epididymitis and prostatitis, respectively^10^. *C*. *pneumoniae* infection can cause pneumonia and respiratory tract infections, however pneumonia is more commonly reported in captive koala populations, and the prevalence appears lower in wild koalas^7,11^. In northern koalas, *C*. *pecorum* is a prevalent pathogen (up to 90%) and severe overt chlamydial disease is commonly observed^1,3^.

**Figure 1:**
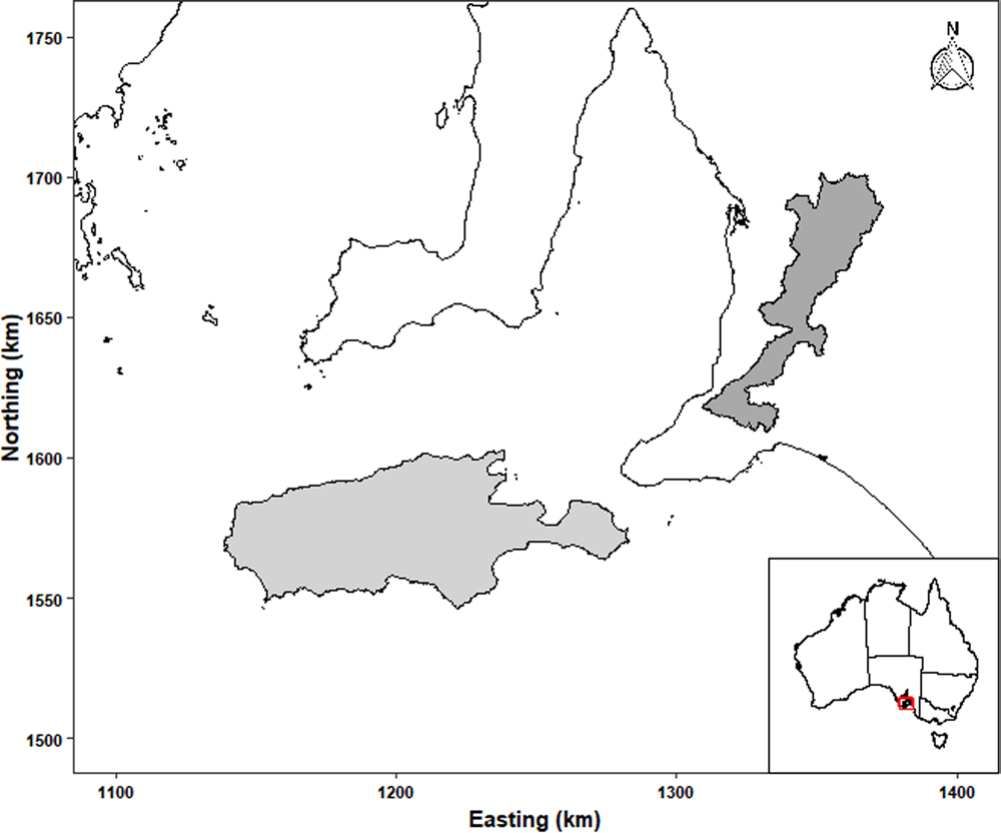
Geographical range of the Mount Lofty Ranges (dark grey) and the Kangaroo Island (light grey) koala populations, South Australia, Australia.

In South Australia, the two largest koala populations^12,13^ are found on the mainland in the Mount Lofty Ranges (MLR) and on Kangaroo Island (KI) (Fig. 1). Although these populations are presumed to be healthy based on the overabundance of koalas within these populations^12,13^, there have been limited reports of the occurrence of infectious diseases.

In MLR, two small scale surveys to investigate *Chlamydia* prevalence were performed two decades ago using conventional PCR; the first reported all six tested koalas to be positive for *Chlamydia*, of unknown species^14^, and in the second 88% (15/17) of animals were *C*. *pecorum* positive with a low incidence of clinical disease reported^3^. The first clinical cases of *C*. *pecorum* disease were only recently reported in three MLR free-ranging koalas that all presented with severe conjunctivitis^15^. Subsequently, a post mortem survey of rescued koalas found a high prevalence of *C*. *pecorum* detection (88%, 57/65) in the MLR. However 28% (n = 16) of positive koalas had no disease associated with infection, 21% (n = 12) showed mild clinical signs of conjunctivitis and/or urinary tract infections and the remaining 51% (n = 29) had inapparent, predominantly microscopic, *C*. *pecorum* infection only^16^.

On KI, previous investigations of *Chlamydia* used outdated techniques with low diagnostic sensitivity and specificity in comparison to PCR, which is regarded as the gold standard^3^. Radiography showed potential paraovarian cysts in eleven female koalas in 1984^17^. Serological studies reported conflicting results, with one study finding no anti-*Chlamydia* antibodies in 63 koalas in 1989^18^ and the second a seroprevalence of 18% in 1997 (n = 201)^19^. The first survey to use direct detection of *C*. *pecorum* DNA was conducted in 1999 and surveyed ten koalas that were all found to be *C*. *pecorum* PCR negative^3^. Given that koalas were introduced into KI from French Island, Victoria in the 1920’s^20^, which was considered *Chlamydia*-free^21^, KI has also been regarded as *Chlamydia*-free. However, *C*. *pecorum* has recently been detected in two French Island koalas^22^ and other reports of introduction of koalas from Queensland to KI in the 1940s^23^ raise doubts on the true status of *C*. *pecorum* in the KI koala population.

At a time when other mainland koala populations are seriously jeopardised by *C*. *pecorum* infection, disease and infertility, MLR koalas appear to have lower levels of overt disease, whilst KI may be the last large *C*. *pecorum*-free population in Australia. Hence this study aimed to determine the prevalence of *C*. *pecorum* in wild ranging koalas from the MLR and KI populations in South Australia and to describe any clinical disease associated with infection.

## Results

### Mount Lofty Ranges targeted survey

**Figure 2:**
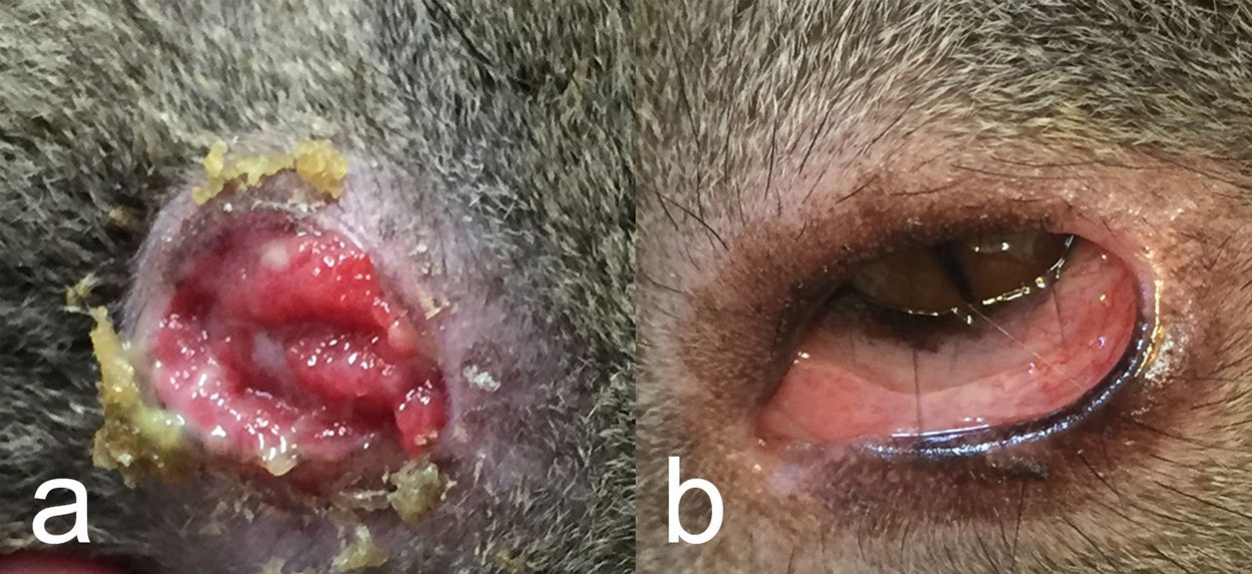
Conjunctival changes in koalas from the Mount Lofty Ranges, South Australia. (**a**) Female koala with unilateral grade 3 severe conjunctivitis, positive for *Chlamydia pecorum*. (**b**) Female koala with grade 1 reddened conjunctiva, negative for *C*. *pecorum* infection.

In the MLR, 30 male and 45 female koalas were captured and sampled. *C*. *pecorum* was detected by qPCR in 46.7% (35/75, Binomial Exact 95% CI: 35.1–58.6%) of koalas. *C*. *pecorum* was more likely to be detected with higher loads at the urogenital site (median, (range)) (34/35; 170 (10–30,600) copies/µL) compared to the ocular site (3/35; 30 (17–2,020) copies/µL). There was no significant difference in prevalence between sexes (females: 55.5%, 25/45; males: 36.7% 11/30; P = 0.156) or between sex and the site of infection (P = 0.339). Despite this, the three koalas that were qPCR positive at the ocular site were all female (3/45). At the urogenital site, females had a higher chlamydial load with median 409 copies/µL (range: 28–30,600 copies/µL) compared to males, 77 copies/µL (range: 10–645 copies/µL). Only 4% (3/75) of koalas presented with overt *C*. *pecorum* clinical disease and all were classified as severe (grade 3). These koalas were all female; one case of mucopurulent pyometra (TWC II), one case of cystitis with urine soiling and scalding of the rump of the koala (TWC VI) and one case of unilateral severe, conjunctivitis (TWC V) (Fig. 2a). Koalas which did not fit the definition of a clinical case included; grade 1 urogenital signs observed as fur discolouration with no scalding of the perineum in koalas qPCR positive (n = 10) and negative (n = 5) for *C*. *pecorum* infection, and grade 1 ocular signs observed in 22 *C*. *pecorum* PCR negative koalas (Fig. 2b).

### Kangaroo Island targeted survey

From the targeted survey, 170 female koalas were sampled over a 3-year period (2014–17) and all found to be *C*. *pecorum* negative by qPCR. The DEW koala program recorded observations for 13,373 individual koalas, surgically sterilised over a 22-year period (1997–2018). None of the clinical records fitted the definition of a clinical chlamydial disease case. Records for two koalas from the targeted survey and 292 koalas from the DEW koala program (all negatives) were not included in the demonstration of freedom analysis as information for age or sex was missing. Data for 10,160 females and 2,921 males were incorporated from the DEW koala program. Details about demographic strata (sex, age class), number of koalas sterilised each year and each surveillance component were reported into the model accessible elsewhere (https://figshare.com/s/590fe1b98c52a4778f83).

### Fecundity of female koalas

Reproductive activity was recorded in female koalas on KI by pregnancy (observed during laparoscopic sterilisation) and the presence of pouch and back young and in MLR by the presence of pouch young, as back young had matured by this time and were not observed. On KI 79.2% (118/149) of sexually mature females were reproductively active, with 41 back young, 66 pouch young and 28 pregnant. Six koalas had both a back and pouch young, and eleven had a back young and were pregnant. The youngest pregnant female was aged 2–3 years (TWC II) and weighed 3.78 kg.

**Figure 3:**
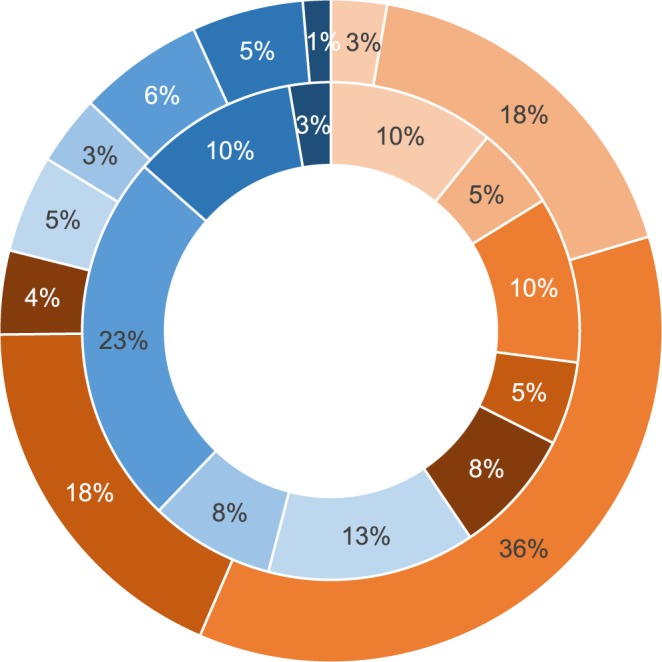
Reproductively active (orange) and inactive (blue) female koalas across age classes (TWC II (lightest) to VI (darkest)) from the Mount Lofty Ranges (inner circle) and Kangaroo Island (outer circle) populations.

In the MLR, reproduction was significantly reduced due to *C*. *pecorum* infection, where only 37.2% (16/43) of female koalas had pouch young. No reproductively active females were infected with *C*. *pecorum*; while females without pouch young were significantly more likely to be infected with *C*. *pecorum* (P < 0.001). In addition, reproductively active females were five times more likely to be infected (RR = 5.0). Of the inactive females, 85.2% (23/27) were infected. Reproductively active females from both populations were more likely to be in excellent body condition (P = 0.040). There was no association between reproductive activity and age in either population (Fig. 3).

### Grade 1 clinical disease observed in KI

In both the targeted survey and the DEW koala program, no KI koalas fitted the definition of a clinical case (>grade 2), however, some koalas were graded 1 for the ocular and urogenital sites. In the targeted survey, 12 (7%) koalas were observed with very mild ocular changes (qPCR negative for *C*. *pecorum*) which were graded with an ocular clinical score of 1. Within the DEW koala program clinical records, 1.08% of koalas (n = 141) were found to have clinical records graded at 1. Ocular changes were recorded in 0.5% of koalas (n = 67), with records such as conjunctivitis (n = 11), corneal scar, ulcer or opacity (n = 35) and periocular inflammation or oedema (n = 21). Urinary tract changes were recorded in 0.03% of female koalas (n = 4), with cystitis (n = 1), haematuria (n = 2) and kidney disease (n = 1) reported. Testicular aplasia was recorded in 0.2% of males (n = 27) and one male with testicular infection. Reproductive changes were recorded in 0.33% of female koalas (n = 43), where records were brief and lacked significant detail. ‘Cystic ovary’ was the most common record (n = 30), and of these koalas 13 were reproductively active; ‘enlarged uterus’ was reported in one female with pregnancy status not recorded; and ‘reproductive adhesions or inflammation’ was also recorded in females with unrecorded pregnancy status (n = 12).

### Demonstration of freedom simulation model

The evidence of absence of *C*. *pecorum* on KI was collected through the 22 year DEW program and targeted surveys including qPCR over a 3-year period. This data supported that the KI koala population is free from *C*. *pecorum* infection in 2018, with an estimated probability of freedom with at least 95% confidence if the prevalence of *C*. *pecorum* on KI was at least 2% and if the yearly probability of introduction of the bacterium in the population is at most 7%. It was not possible to reach a minimum of 95% probability of freedom if the design prevalence was 1% or less. If the design prevalence is >2%, the probability of freedom was at least 95% regardless of the probability of introduction (Table 1).

**Table 1:** Sensitivity analysis of the probability of freedom (*P*_*free*_) (mean, lower and upper limits) after 22-years of surveillance for given design prevalence (P*) and probability of introduction (*P*_*intro*_) values. Lower *P*_*free*_ limit above a 95% confidence estimate (bold), and above 99% confidence estimate (bold, italicised).

Design Prevalence	Yearly probability of introduction			
	1%	7%	10%	20%
1%	96.5% (89.4–99.1%)	84.8% (66.5-93.5%)	76.6% (50.6–90.4%)	38.9% (9.4–78.2%)
2%	***99.5% (99.3–99.6%)***	**96.1% (95.1–97.4%)**	94.3% (92.8–96.1%)	87.7% (84.3–91.6%)
5%	***99.9% (99.8–99.9%)***	**99.0% (98.5–99.6%)**	**98.5% (97.8–99.4%)**	**96.7% (95.3–98.6%)**
10%	***99.9% (99.9–100%)***	***99.8% (99.7–99.9%)***	***99.8% (99.6–99.9%)***	***99.5% (99.1–99.9%)***
15%	***99.9% (99.9–100%)***	***99.9% (99.9–100%)***	***99.9% (99.9–100%)***	***99.9% (99.8–100%)***

**Figure 4:**
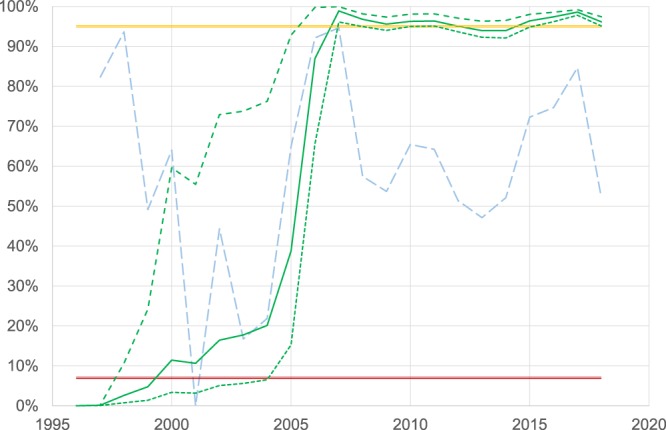
Demonstration of freedom from *Chlamydia pecorum* infection in the KI koala population. Mean estimates for surveillance sensitivity (*SSe*) and probability of freedom, *P*(*free*)_*i*_ from *C*. *pecorum* between 1997 and 2018. The mean *P*(*free*) (solid, dark green line) with 95% CI as lower (shorted, dashed, dark green line) and upper (long, dashed, dark green line) confidence limits and *SSe* (long, dashed, light blue line) with a probability of introduction, *P*(*intro*) of 7% (solid double red line) and a design prevalence (P*) of 2%, concluded in 2018 above the 95% limit (solid double yellow line).

The evolution over time of the sensitivity of the surveillance (*SSe*_*i*_) and the probability of freedom (95% CI), with a 2% design prevalence and a risk of introduction at 7% is represented in Fig. 4. The *SSe* displayed is the average of 10,000 iterations in the model. The *SSe* was highly depended on the surveillance effort (i.e. the number of koalas screen and accuracy of detection). Over the first two years (1997–1998), the *SSe* was above 80%, but dropped to below 70% from 1999 to 2004 due to a smaller number of records for each year (maximum 564) and no koalas captured in 2001, reflecting the *SSe* to 0%. From 2005–2018, the *SSe* remained above 50%. The highest *SSe* was above 90% in 2006 and 2007 with the largest number of koalas observed in the DEW program, with 1,522 and 1,705 observed, respectively. The targeted survey from 2015 to 2017 increased the *SSe* to above 80% from below 70% in 2014 with a similar number of koalas captured.

The lower limit of the *P*(*free*)_*i*_ was below 90% up until 2007. In 2007, with the large number of koalas captured, minimum *P*(*free*)_*i*_ increased to 96.0% and approximated 95% with the last *P*(*free*)_2018_ of 95.1% (Fig. 4).

## Discussion

*Chlamydia pecorum* is a pathogen of critical, national importance to the conservation of the koala species. Koala populations in northern Australia (Queensland and New South Wales) are experiencing significant declines that have been attributed to a number of factors, including deforestation, urbanisation, trauma (vehicular and dog attacks)^1,24^ and *C*. *pecorum*^25^. In the present study, we have shown that *C*. *pecorum* is well established in the wild Mount Lofty Ranges (MLR) population, with 46.7% of the surveyed koalas positive by PCR, but clinical disease was only observed in 4% of koalas. In KI koalas, the population was demonstrated to be free of *C*. *pecorum* infection with 95% confidence.

In wild MLR koalas, the prevalence of *C*. *pecorum* infection was found to be high, but was half of the prevalence reported in the previous study of necropsied koalas from the same area^16^. Overt *C*. *pecorum* disease was only observed in three koalas, which is substantially less than that seen in northern populations, where 20.8% (5/24) of free-ranging koalas from Queensland populations presented with overt *C*. *pecorum* disease^26^. Urogenital infections (2/3) were more common than ocular infections (1/3), which is also the trend reported in Victorian koalas^27^. Although *C*. *pecorum* infection was reported in the MLR population two decades ago^3,14^, clinical disease associated with infection is only recently being reported more frequently with increasing disease severity^15,16^. Prior to 2012, overt clinical disease was not observed by local veterinarians treating rescued koalas from the MLR population (N. Speight pers. comm.). The increasing prevalence may be due to increased awareness of chlamydial disease but may also suggest a possible change in pathogenicity of *C*. *pecorum* or increased host susceptibility. Possible causes include the introduction of a new pathogenic variant of *C*. *pecorum*^28^; an upregulation in the transmission of a virulence plasmid, *pCpec*^29^; or the recent introduction of the Koala retrovirus^30^, which may predispose koalas to develop clinical disease through immunosuppression^31,32^. Evidence has shown immunologically naïve koalas exposed to *C*. *pecorum* are at risk of high morbidity and mortality^33–35^. The implications for *C*. *pecorum* infection in the MLR population is unknown, but chlamydial infection was found in this study to significantly reduce female koala fecundity. Hence monitoring of the MLR population should continue to detect any future changes in chlamydial prevalence and pathogenicity that could cause the population to decline similarly to that observed in northern koala populations.

In KI, the model has demonstrated that the DEW koala program and targeted survey had sufficient surveillance sensitivity to detect an established *C*. *pecorum* infection in the KI population if the prevalence was greater than 2%. Hence it remains possible that *C*. *pecorum* may be present in the population at a very low prevalence, for instance if it had been recently introduced. The risk of introduction of *C*. *pecorum* into KI is unknown. Possible routes of introduction include from infected koalas, domestic livestock or other native species. *C*. *pecorum* is thought to transmit between koalas by direct contact^3,35^, which is likely to include sexual transmission^26^ and mother to offspring^27,36^. As the introduction of infected koalas to KI is unlikely to occur due to strict DEW regulations on koala care and movement, koala faeces infected with *C*. *pecorum*^37^ may pose a threat, based on what is known in other species, as the faecal-oral route is thought to be the most likely route of transmission between domestic cattle (*Bovis taurus*) infected with *C*. *pecorum*^38^.

This highlights the potential introduction of *C*. *pecorum* into KI through livestock. While the koala has a distinct clade of *C*. *pecorum* isolates which infect and cause disease in the koala, there are some genetically similar strains of *C*. *pecorum* shared between domestic cattle, sheep (*Ovis aries*) and the koala^39,40^. The recently discovered *C*. *pecorum* isolates in French Island koalas were found to be genetically related to livestock *C*. *pecorum* genotypes^22^, suggesting potential transmission between species. Sheep farming is a common activity on KI, with up to 680,000 sheep^41^, and while the presence of *C*. *pecorum* in KI sheep remains unknown, *C*. *pecorum* has been found in sheep in south-eastern South Australia^42^. Investigation into the presence of *C*. *pecorum* in livestock on KI is underway.

Transmission may occur either directly by exposure of koalas to infected livestock faeces when they travel on the ground between trees, or via an intermediate species such as has been hypothesised with possums^43^. It has been found that the mountain brushtail possum (*Trichosurus caninus*) in Victoria can be infected with *C*. *pecorum*^44^ and due to its arboreal nature, it may introduce contaminated livestock faeces into the eucalypt trees on which koalas feed. While this species of possum is not present on KI, the common brushtail possum (*Trichosurus vulpecula*) is highly abundant and has also been shown to share some intestinal parasites with sheep on KI^45^. To ensure trans-species transmission does not occur with the koala on KI, *C*. *pecorum* in domestic livestock and native species on KI should be investigated through targeted surveillance, in conjunction with increased biosecurity measures implemented to ensure that *C*. *pecorum* is not brought onto the island.

The DEW sterilisation program on KI provides a sensitive surveillance tool for both ocular and urogenital disease. Review of the clinical data from the KI DEW koala program found 141 koalas (1.08%) with possible signs of chlamydial disease that were assigned a grade 1 chlamydial score. This was due to minimal details of clinical signs recorded, that no diagnostic PCR performed to confirm *C*. *pecorum* infection and that although these changes were consistent with chlamydial infection, they were not pathognomonic for *C*. *pecorum*, and could be explained by other causes. Ocular and urinary tract disease has been reported in southern Australian koalas without *C*. *pecorum* infection^16,21,27^, and with recent analysis of the ocular and urogenital microbiomes in the koala, there are possibly other pathogens which cause similar clinical signs^46,47^. Kidney disease may be due to oxalate nephrosis, which is highly prevalent in the MLR population^48,49^, while testicular aplasia has been reported in association with reduced genetic diversity in KI koalas^50^. A common report was “ovary cyst” with no additional description of the cysts, such as size or quantity. As some koalas were also reproductively active, it cannot be determined if these described cysts were pathological^9^, or a result of normal reproduction, such as large follicles^51^.

The impacts of *C*. *pecorum* on the eastern Australian koala populations are devastating with considerable morbidity and mortality as a result of infection and declines in population numbers due to infertility. We have estimated freedom from infection on KI over a 22-year period with >95% confidence. Hence this large, isolated *C*. *pecorum*-free population of koalas holds significant importance as insurance for the future of the species. Every effort should be made to ensure the population remains *C*. *pecorum*-free so that these koalas could be used, in conjunction with the newly developed *C*. *pecorum* vaccine^52^, to re-populate declining populations, and may ultimately ensure the survival of the koala for generations.

## Materials and Methods

### Chlamydia pecorum targeted surveys

In the MLR, 75 wild koalas were captured in April 2016 using ropes and poles as described previously^19^, and relocated to a nearby sampling site. On KI, 170 wild koalas were sampled between November 2014 and February 2017 in conjunction with the South Australian Department for Environment and Water (DEW) Koala Sterilisation Program. The Koala Sterilisation Program was implemented in 1996 to monitor the overabundant KI population and uses surgical sterilisation as a means for population management^53^. These surveys were approved by The University of Adelaide Animal Ethics committee (S-2013-198, S-2015-138) with State Government DEW Scientific Research permits (Y26054-6, U26431-1) and completed in accordance with the University of Adelaide and State Government guidelines and regulations.

For clinical examination, MLR koalas were anaesthetised with either alfaxalone (Alfaxan, Jurox, United Kingdom) (3.5 mg/kg) IM or alfaxalone (2 mg/kg) with medetomidine (Domintor, Vetquinol, United Kingdom) (40 µg/kg) IM, and on KI, isoflurane (2–5%) and oxygen (2%/min) was used. Each koala was aged by the degree of wear of the upper premolar (Tooth wear class (TWC) I, 1–2 years; II, 2–3 years; III, 4 years; IV, 5–6 years; V, 10–12 years; VI, 12+ years^54^). For KI females, laparoscopic sterilisation was performed allowing visualisation of the reproductive tract for pregnancy or any pathological changes. Reproductive activity was recorded in MLR females by the presence of pouch young and on KI by pregnancy, pouch and/or back young. Koalas were classified as sexually immature if their bodyweight <3.90 kg^19^. Each koala was graded for ocular and urogenital clinical signs consistent with *C*. *pecorum* using a 4 scale system (grade 0, no disease; 1, mild disease; 2, moderate disease; 3, severe disease)^55^. Two dry aluminium shaft swabs (Copan Italia, Brescia, Italy) of the conjunctiva and cloaca were collected^7^ and stored at −80 °C until *C*. *pecorum* detection.

### Historical clinical examination on KI

The DEW koala program collected clinical examination data during routine surgical sterilisations of koalas conducted every year from approximately November to March for 22 years (1997–2018). The clinical data included sex (male or female), age class (as described above) and signs of disease. Individual clinical records were reviewed retrospectively and ocular and urogenital findings consistent with *C*. *pecorum* infection were graded as described above^55^. A koala was classified as a positive clinical case if the clinical record described grade 2 disease or above for either the ocular or urogenital sites. This case definition favours the specificity of the classification to minimise possible false positive cases from mild, non-pathognomonic clinical signs^46,48,50,51^.

### *Chlamydia pecorum* molecular detection

Ocular and urogenital swabs from individual koalas were screened for *C*. *pecorum* detection. DNA was extracted using Qiagen DNA Mini kit (Qiagen, Hilden, Germany) and extracted DNA was pooled and amplified using a *C*. *pecorum*-specific qPCR targeting a 209 bp fragment of C. pec HP gene^56^. Positive pooled swabs were re-tested separately. Briefly, the qPCRs were performed in a final volume of 20 µl, including 10 µl iTaq master mix (Bio-Rad, California, USA), 1 µl of 10 µM each of forward and reverse primer (Sigma-Aldrich, Australia), 3 µl dH_2_O and 5 µl template DNA. Cycling conditions consisted of 15 min at 95 °C, followed by 35 cycles of 15 sec at 94 °C, 15 sec at 57 °C and 30 sec extension at 72 °C. Samples were tested in duplicates, and negative control (dH_2_O) and positive control (*C*. *pecorum* Marsbar DNA) were included in each assay. In each assay, infectious load was quantified by plotting the crossing points against a standard curve produced using a serial dilution from 10^6^ to 10^0^ copies/µl of the known standard, *C*. *pecorum* target amplicon. C. pec HP gene amplicon was characterised with a high-resolution melt (HRM) of 77.5 ± 0.5 °C. DNA quality was assessed by detecting a 122 bp fragment of the koala β–actin gene. The reaction was performed in 25 μl containing 5 μl of 5X Taq polymerase buffer (Bioline, Australia), 2 mM of magnesium chloride, 0.1 mM of dNTP mix, 1 mM of each of the published β-actin primers, 5′-AGATCATTGCCCCACCT-3′ (sense) and 5′-TGGAAGGCCCAGATTC-3′ (anti-sense)^57^, 0.25 µL of MyTaq DNA polymerase (Bioline, Australia), and 10 µL of DNA template. The PCR conditions were performed as recommended by the Bioline PCR kit, with a 58 °C primer annealing temperature and final extension at 72 °C for 10 minutes.

#### Univariate analysis

Statistical analysis was performed using SPSS v.24 to determine significance based on sex, age and *C*. *pecorum* status (α = 0.05). For continuous *Chlamydia* load variables, a Shapiro-Wilk test was performed to determine Gaussian distributions. For variables with normal distribution, an F-test was performed to determine equal variance prior to a two-way independent t-test. For non-parametric variables a Kruskal-Wallis H analysis was performed with post-hoc Mann-Whitney U test. Chi-squared analysis was performed to determine relationships and odds ratio between *C*. *pecorum* infection, sex, age and reproduction status.

### Demonstration of freedom from *Chlamydia pecorum* on KI

The probability that the KI koala population is *C*. *pecorum*-free was estimated by collating qPCR results from the targeted survey and DEW koala program historical clinical data in a scenario tree modelling approach^58^. The probability of freedom of a given year of surveillance *i* (*P*(*free*)_*i*_) was calculated using the Bayesian approach from the probability of freedom from the prior year *i* − *1* (*P*(*free*)_*i−1*_) and the surveillance system sensitivity (*SSe*_*i*_) and specificity (*SSp*_*i*_), reflecting the strength of the evidence collected during the year *i*^58^:

1 $$P{(free)}_{i}=\frac{Prob{(free)}_{i-1}\times SS{p}_{i}}{Prob{(free)}_{i-1}\times SS{p}_{n}+(1-P{(free)}_{i-1})\times (1-SS{e}_{i})}$$

*P*(*free*)_*i*_ was then adjusted for the probability of introduction (*P*(*intro*)_*i*_) during the same year *i*^58^:

2 $$adjusted\,P{(free)}_{i}=1-((1-P{(free)}_{i})+P{(intro)}_{i}-(1-P{(free)}_{i})\times P{(intro)}_{i}$$

### Surveillance System Specificity (*SSp*)

The diagnostic specificity of both diagnostic methods, qPCR and clinical examination, were perfect (100%) at the individual animal level. Therefore, the *SSp* relying on these two methods at the population level was deduced as also 100%. This deduction was further supported by the fact that none of the surveyed KI koalas were classified as positive (i.e. no potential false-positive).

### Surveillance System Sensitivity (*SSe*)

The *SSe* was calculated based on 12 month periods to match the yearly cycle of the surveillance. In a given year *i*, the *SSe*_*i*_ was calculated from the sensitivity of the *C*. *pecorum* targeted survey (*CSe*^*Survey*^_*i*_) and DEW koala program (*CSe*^*DEW*^_*i*_) surveillance components as follow^58^:

3 $$SS{e}_{i}=1-((1-CS{e}_{i}^{Survey})\times (1-CS{e}_{i}^{DEW}))$$

### System Component Sensitivity (CSe)

In a given year, *CSe*_*i*_ was calculated from the component unit sensitivity (*CSeU*_*i*_, probability of detecting a single infected koala if sampled at a given period) of each surveillance component as follow^58^:

4 $$CS{e}_{i}=1-{(1-CSe{U}_{i})}^{n}$$

where *n* is the total number of koalas sampled during a given activity and given year.

### Component Unit Sensitivity (CSeU)

**Figure 5:**
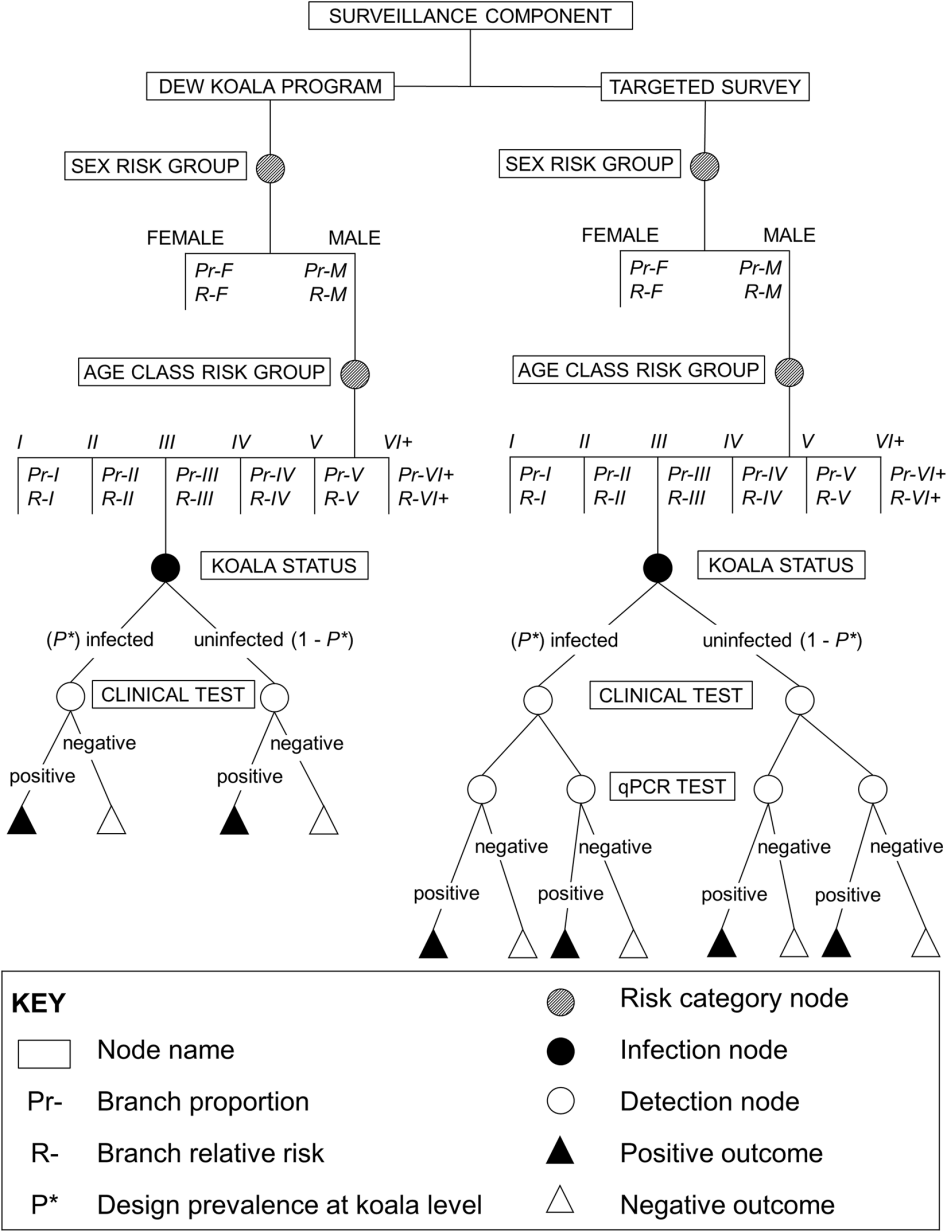
Structure of scenario trees used to estimate the Component Unit Sensitivity to detect *Chlamydia pecorum* infection in individual koalas during the DEW Koala Program or *C*. *pecorum* Targeted Survey, respectively.

For each year *i*, the *CSeU*_*i*_ was calculated from scenario trees representing all possible scenarios, including risk categories (sex and age class) and detection outcomes (positive or negative)^59^. Two separate trees were built for each surveillance component, the targeted survey and DEW koala program (Fig. 5). The probability of each scenario tree branch was calculated by multiplying the representation of its risk category class in the total sample, the adjusted risk (AR) of the risk category class, the design prevalence (assumed minimum prevalence of *C*. *pecorum* when the infection is established in a koala population) (P*) and the probability of detection according to the diagnostic method accuracy (i.e. diagnostic sensitivity and specificity, *DSe* and *DSp* respectively).

### Adjusted Risk (AR)

For each risk category classes (sex and age classes), an AR_ageXsex_ was calculated by multiplying the AR for sex and the AR for age class. The AR for sex were calculated using the relative risk of *C*. *pecorum* infection (RR) of males compared to females and the assumed population representation (PrP) of each sex as follow^58^:

5 $$A{R}_{Female}=\frac{1}{(R{R}_{Male}\times Pr{P}_{Male})\times Pr{P}_{Female}}$$

6 $$A{R}_{Male}=A{R}_{Female}\times \,R{R}_{Male}$$

The AR for the six age classes were calculated similarly using the relative risk (RR) of each age class compare to the first age class (TWC I) and the assumed population representation (PrP) of each class for a given sex^58^:

7 $$A{R}_{TWC1/sex}=\frac{1}{{\prod }_{2}^{6}(R{R}_{TWCJ/sex}\times Pr{P}_{TWCJ/sex})\times Pr{P}_{TWC1/sex}}$$

8 $$A{R}_{TWCJ/sex}=A{R}_{TWC1/sex}\times \,R{R}_{TWCJ/sex}$$

where *J* is the age class ranging from TWC II to VI.

### Diagnostic sensitivity and specificity (*DSe* and *DSp*)

*DSe* and *DSp* of the qPCR (DSe_PCR_ and DSp_PCR_) were estimated in-house by comparing the proportions of positive and negative results when screening known positive and negative standards respectively. The *DSe* and *DSp* of the clinical examination (DSe_Clin_ and DSp_Clin_) were deduced by crossing the results from the clinical examination with the qPCR from koalas surveyed in the MLR (population infected with *C*. *pecorum*). DSe_Clin_ and DSp_Clin_ were calculated using the following formulae^60^:

9 $$DS{e}_{Clin}=\frac{{n}_{1}DS{p}_{PCR}-c}{nDS{p}_{PCR}-{m}_{0}}$$

10 $$DS{p}_{Clin}=\frac{{n}_{0}DS{e}_{PCR}-b}{nDS{e}_{PCR}-{m}_{1}}$$

where *n* is the total number of koalas tested in the MLR; *n*_*1*_ is the count of individuals with clinical signs present (positive clinical cases); *n*_0_ is the count with no clinical signs (negative clinical cases); *b* is the count of PCR positive koalas without clinical signs; *c* is the count of PCR negative koalas with clinical signs; *m*_1_ is the total count of PCR positive and *m*_0_ is the total count of PCR negative koalas.

### Model stochasticity

To account for the uncertainty about model parameters, stochasticity was built into the model by allocating a distribution to the parameters using the PopTools Excel add-in v3.2 (PopTools 2011). Distribution used in the model and the source of the information used to parametrise the distribution are reported in Table 2. Probability parameters were allocated Beta distribution parametrised with BetaBuster v1.0 software (Su 2012 https://www2.vetmed.ucdavis.edu/cadms/local_resources/docs/betabuster012006.zip) according to their exact Binomial 95%CI. Other model parameters were allocated a Pert distribution according to their reported 95% CI. The simulation was run for 10,000 iterations and the model outputs’ distributions were reported with the mean, 2.5^th^ and 97.5^th^ percentiles. A population of koalas was deemed free from the infection if the 2.5^th^ percentile (lower limit) the estimated probability of freedom was >95%.

**Table 2:** Model parameters respective reported uncertainty (estimate sand 95% CI), allocated distribution for the stochastic modelling and source of information.

Model parameters	Estimate	95%CI lower limit	95%CI upper limit	Distribution	Source
**Population proportion**					
PrP_Female_	50.7%	54.4%	47.1%	Beta (365.9257, 355.3342)	55
PrP_TWC I/Female_	38.8%	33.9%	43.9%	Beta (138.1517, 217.4571)	55
PrP_TWC II/Female_	15.6%	12.1%	19.6%	Beta (52.5587, 280.6404)	55
PrP_TWC III/Female_	22.2%	18.1%	26.7%	Beta (76.5331, 266.265)	55
PrP_TWC IV/Female_	17.9%	14.2%	22.2%	Beta (61.1473, 276.0853)	55
PrP_TWC V/Female_	4.0%	2.2%	6.4%	Beta (15.8344, 360.9804)	55
PrP_TWC VI/Female_	1.6%	0.6%	3.4%	Beta (6.9566, 371.3016)	55
PrP_Male_	49.3%	45.6%	52.9%	Beta (354.923, 365.5023)	55
PrP_TWC I/Male_	44.3%	39.1%	49.5%	Beta (156.1654, 196.1467)	55
PrP_TWC II/Male_	13.9%	10.5%	17.8%	Beta (44.9898, 274.427)	55
PrP_TWC III/Male_	20.4%	16.4%	24.9%	Beta (67.8923, 262.326)	55
PrP_TWC IV/Male_	14.7%	11.2%	18.7%	Beta (47.83, 273.3078)	55
PrP_TWC V/Male_	3.0%	1.5%	5.3%	Beta (11.8921, 354.4976)	55
PrP_TWC VI/Male_	3.8%	2.1%	6.3%	Beta (14.8459, 351.1047)	55
**Relative risk**					
RR_Female_^a^	1.00	—	—	—	27
RR_Male_	1.52	1.08	2.15	Pert (1.52, 1.08, 2.15)	27
RR_TWC I_^b^	1.00	—	—	—	27
RR_TWC II_	1.00	—	—	—	27
RR_TWC III_	1.29	0.74	2.25	Pert (1.29, 0.74, 2.25)	27
RR_TWC IV_	1.29	0.74	2.25	Pert (1.29, 0.74, 2.25)	27
RR_TWC V_	2.77	1.44	5.37	Pert (2.77, 1.44, 5.37)	27
RR_TWC VI_	2.77	1.44	5.37	Pert (2.77, 1.44, 5.37)	27
**Test accuracy**					
DSe_PCR_	100.0%	83.9%	100.0%	Beta (21.00000977, 1)	Jelocnik, unpublished
DSp_PCR_	100.0%	76.8%	100.0%	Beta (14.000000485, 1)	Jelocnik, unpublished

PrP, expected proportion of sex and age within sex, of the population.

TWC, tooth wear class.

RR, relative risk.

DSe_PCR_, probability of qPCR to test positive if the koala is truly infected.

DSp_PCR_, probability of qPCR to test negative if the koala is truly non-infected.

^a^Sex risk of *C*. *pecorum* in a koala relative to the risk of *C*. *pecorum* in females.

^b^Age risk of *C*. *pecorum* in a koala relative to the risk of a koala with *C*. *pecorum* in TWC I.

### Sensitivity Analysis

No robust estimates of the design prevalence (P*) and the probability of introduction (*P*(*intro*)) could be sourced. A sensitivity analysis was conducted by varying both parameters and assessing the impact of the final probability of freedom estimate and 95% CI. As the risk of introduction may vary with time, we assumed in the model that the probability of introduction was at its maximum possible value for the entire surveillance period.

The model to assess freedom from *C*. *pecorum* was implemented in MS Excel (2013) and a copy is accessible online (https://figshare.com/s/590fe1b98c52a4778f83).

## Data Availability

The dataset generated and analysed during this study is available in the Figshare repository [https://figshare.com/s/590fe1b98c52a4778f83]. Further data generated during this study is available from the corresponding author on reasonable request.
